# Differential Regulation of CD45 Expression on Granulocytes, Lymphocytes, and Monocytes in COVID-19

**DOI:** 10.3390/jcm11144219

**Published:** 2022-07-20

**Authors:** Muhammad G. T. Ahmed, Andreas Limmer, Christoph Sucker, Khaled Mohamed Fares, Sahar Abdel-Baky Mohamed, Ahmed H. Othman, Marc Moritz Berger, Thorsten Brenner, Matthias Hartmann

**Affiliations:** 1 Department of Anesthesiology and Intensive Care Medicine, University Hospital Essen, University Duisburg-Essen, 45147 Essen, Germany; muhammadthabet87@aun.edu.eg (M.G.T.A.); andreas.limmer@uk-essen.de (A.L.); marc.berger@uk-essen.de (M.M.B.); thorsten.brenner@uk-essen.de (T.B.); 2 Medizinisches Versorgungszentrum (MVZ), COAGUMED Gerinnungszentrum, 10789 Berlin, Germany; cs@coagumed.de; 3 Medizinische Hochschule Brandenburg (MHB), 14770 Brandenburg an der Havel, Germany; 4 Anesthesia, Intensive Care and Pain Management, South Egypt Cancer Institute, Assiut University, Assiut 7111, Egypt; faressali@yahoo.com (K.M.F.); drsaher2008@yahoo.com (S.A.-B.M.); ahmadhothman@gmail.com (A.H.O.)

**Keywords:** COVID-19, CD45, inflammation, lipopolysaccharides, granulocytes, lymphocytes, monocytes

## Abstract

CD45 is a transmembrane glycoprotein and protein tyrosine phosphatase expressed on the surface of all nucleated hematopoietic cells. While there is increasing evidence demonstrating the involvement of CD45 in immune system regulation, no information on CD45 expression in inflammation and sepsis is currently available. Therefore, we determined the CD45 surface expression on granulocytes, lymphocytes, and monocytes in patients with COVID-19 and healthy volunteers in both absence and presence of lipopolysaccharide (LPS). Following approval by the local ethics committee, whole blood samples were obtained from patients with COVID-19 infection on day 1 of hospital admission and healthy volunteers. Samples were incubated in absence and presence of LPS and CD45 was measured in granulocytes, lymphocytes, and monocytes using flow cytometry. In comparison with healthy individuals, COVID-19 patients showed an increased CD45 expression on the surface of granulocytes (+35%, *p* < 0.02) and lymphocytes (+39%, *p* < 0.0001), but a reduced CD45 expression on monocytes (−35%, *p* < 0.0001). LPS incubation of whole blood from healthy individuals increased the CD45 expression on granulocytes (+430%, *p* < 0.0001), lymphocytes (+32%, *p* = 0.0012), and monocytes (+36%, *p* = 0.0005), respectively. LPS incubation of whole blood samples from COVID-19 patients increased the CD45 expression on granulocytes and monocytes, and decreased the CD45 expression on lymphocytes. In conclusion, CD45 expression on leucocytes is altered: (1) in COVID-19 patients, and (2) in in vitro endotoxemia in a complex cell-specific way, thus representing a new immunoregulatory mechanism.

## 1. Introduction

CD45 (common leucocyte antigen) is a transmembrane glycoprotein with a molecular weight of 180–220 kDa expressed on all leucocytes and constitutes about 10% of cell surface antigens [1,2,3]. CD45 is highly conserved and exists not only in mammals, but also in chicken, sharks, and mosquitos [4]. Six alternatively spliced isoforms differing only in the extracellular portion of the molecule have been described [1,5,6]. However, among these variants, no differences in function have been described yet. CD45 is a receptor protein tyrosine phosphatase and some extracellular CD45 ligands have been identified. However, most of them do not bind exclusively to this receptor, or are present only under distinct conditions, such as infection or pregnancy [1]. Placental protein 14, and pUL11, a transmembrane protein from cytomegalovirus (CMV RL11), have both been demonstrated to disrupt T-cell receptor signaling and to inhibit T-cell proliferation via CD45 [7,8,9]. Due to the absence of specific ligands in healthy humans, it is speculated that CD45 is in a constitutive active state without the presence of any ligand and thus the activation by receptor ligands is not required for its physiological functions [10]. 

Concerning the substrates of CD45, a broad phosphoprotein substrate spectrum has been shown in vitro [11,12]. In vivo, cell-type-dependent dephosphorylation of a number of Src family kinases and Src family kinase substrates, as well as Janus kinases, has been demonstrated [10].

Several immune system functions have been described as being modulated by CD45. In T cells, defects of development and function have been described in CD45-deficient mice, highlighting the dependency of T-cell function on CD45 [13]. Similarly, reduced B-cell function has been demonstrated in CD45-deficient cell lines [10,14,15]. Furthermore, CD45 has been described as affecting Toll-like receptor signaling in macrophages and bone-marrow-derived dendritic cells leading to the production of cytokines, the adhesion of T cells and macrophages, and the increase chemotaxis in neutrophils and T cells [10]. In humans, two gene polymorphisms have been detected in the PTPCR gene, C77G, and A138G. The former is associated with immune disorders including autoimmune hepatitis, HIV infection, and multiple sclerosis, while A138G is associated with hepatitis B, Graves´ disease, and severe combined immunodeficiency [16,17,18]. Furthermore, CD45 has been shown to be involved in tumor-induced immunosuppression in peripheral blood mononuclear cells [19].

Although the evidence for the critical involvement of CD45 in immune regulation is steadily increasing, no information on the effect of endotoxemia and inflammatory diseases on the surface expression of CD45 on leucocyte subtypes is available. 

In an initial experiment, we demonstrated that LPS increases CD45 expression of granulocytes, lymphocytes, and monocytes. We therefore decided to study: (1) the ex vivo effect of LPS in volunteers, (2) the CD45 expression in an inflammatory disease (COVID-19), and (3) the eventual differences in LPS-induced CD45 expression in volunteers and COVID-19 patients. 

## 2. Materials and Methods

The study was performed after approval by the local ethics committee (17-7824-BO and additional amendment). In a first series, the effect of LPS (incubation for 0, 30, 120, 240, and 360 min) on the CD45 expression in whole blood samples from 6 volunteers was investigated and the time course of CD45 surface expression was determined in whole blood samples from healthy volunteers. In a second series, blood was drawn from 112 patients with COVID-19 at day 1 of hospital admission and 20 healthy volunteers, respectively. Disease severity was judged from the medical records according to a recently published score [20]. Based on the results of the first series already showing increased CD45 expression after 30 min, blood was incubated with LPS or vehicle for 60 min in the following series. Two mL of blood were drawn in lithium–heparin tubes and aliquots were incubated with LPS (100 ng/mL, Escherichia coli O111:B4, Sigma-Aldrich, St. Louis, MO, USA) or vehicle in a final volume of 50 µL at 37 °C for the indicated time interval. Thereafter, samples were incubated with antibodies directed against CD45 (PerCP/Cyanine5.5 anti-human CD45, isotype mouse IgG1, κ from Biolegend (San Diego, CA, USA) 3 µL) for 10 min at room temperature (1 µg/mL final concentration). Then, erythrocytes were lysed by incubation with 0.08 mL RBC lysis buffer (from pluriselect life science) for 10 min at 4 °C. Finally, samples were subjected to a flow cytometer (CytoFlex Flow Cytometer, Beckman Coulter, Inc. (Brea, CA, USA) PN B49006AE, November 2015). 

For the detection of leucocyte subtypes, gates were defined by use of the forward and sideward scatter, as well as CD45 expression (PerCP/Cyanine5.5 fluorescence intensity). For the evaluation of the results, mean CD45 fluorescence intensity (MFI) of granulocytes, lymphocytes, and monocytes was determined. For analysis of raw data, the device´s software was used (CytExpert version 2.4.0.28 Beckman Coulter, Inc.). Daily quality checks of the flow cytometer were made using cytoflex fluorospheres from Beckmann Coulter to guarantee the long-term reproducibility of measurements. For the statistical evaluation and graphs, the software Prism was used (version 8.4.3, GraphPad software, San Diego, CA, USA). Flow cytometry data are given as mean and standard error of the mean. Student’s *t*-test was used for statistical evaluation. Moreover, receiver operating characteristic (ROC) curves, area under the curve (AUC), and asymptotic significance levels were generated to evaluate the ability of CD45 measurements to discriminate between volunteers and COVID-19 patients. 

## 3. Results

From the 112 COVID-19 patients included in the study, 45 patients were female. The median age was 64 years with a range of 19–88 years. Mean disease severity according to the WHO classification was 4 with a range from 4 to 8. Six patients in the cohort died. 

**Figure 1:**
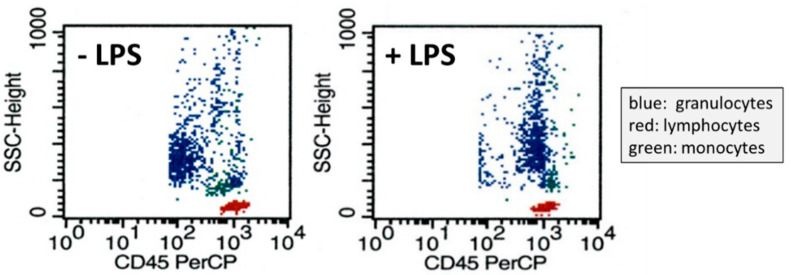
Original diagrams from FACS analysis showing the CD45 surface expression of leucocytes subsequent to 4 h incubation with vehicle (**left**) and lipopolysaccharide (LPS, **right**). Shown are fluorescence of leucocytes caused by peridinin–chlorophyll–protein complex marked antibodies directed against CD45 on the abscissa and the sideward scatter on the ordinate. Note the marked increase in CD45 on the surface of granulocytes in LPS-treated cells.

**Figure 2:**
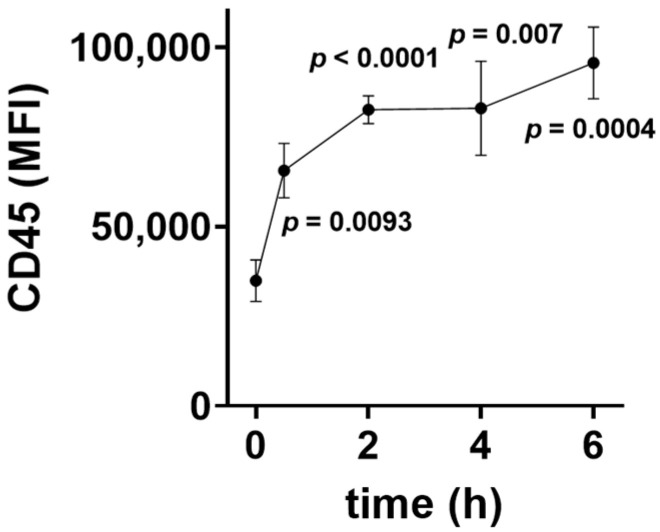
Time course of the lipopolysaccharide (LPS)-induced CD45 surface expression on human granulocytes in 6 volunteers. Whole blood samples were incubated with 100 ng/mL LPS for various time intervals; CD45 surface expression was determined with cell cytometry and labeling of cells with fluorescent CD45 antibodies. Values are given as mean and SEM. *p*: Student’s *t*-test significance level of the difference of mean fluorescence intensity between t = 0 and the respective time point.

In Figure 1, the effect of LPS added to whole blood samples on CD45 surface expression on granulocytes is shown. In the absence of LPS, most granulocytes showed a low CD45 expression and only some granulocytes showed a high expression of the surface marker. Incubation with LPS led to a shift from a low CD45 phenotype to a high CD45 phenotype with a fourfold increase in CD45 surface expression. The time course of CD45 surface expression induced by LPS (100 ng/mL) in healthy volunteers is shown in Figure 2, revealing the rapid onset of the protein tyrosine phosphatase expression in granulocytes starting 30 min after incubation with LPS (100 ng/mL).

**Figure 3:**
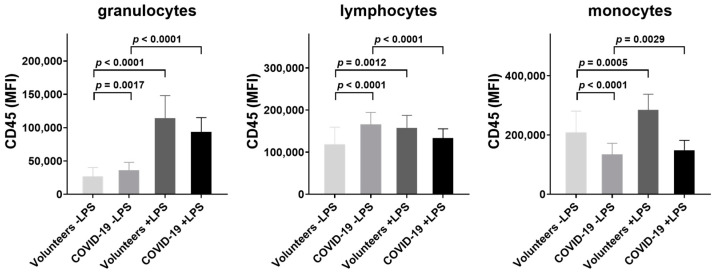
Surface expression of CD45 on granulocytes, lymphocytes, and monocytes in healthy volunteers (*n* = 20) and patients with COVID-19 (*n* = 112). Whole blood samples were incubated in absence and presence of LPS (100 ng/mL, 60 min), stained with a fluorescent CD45 antibody, and subjected to FACS analysis. Data are given as mean and SEM; Student’s *t*-test was used for statistical evaluation.

CD45 surface expression in 20 healthy volunteers and 112 COVID-19 patients on granulocytes, lymphocytes, and monocytes in both absence and presence of LPS are summarized in Figure 3. Comparison of CD45 expression on granulocytes, lymphocytes, and monocytes from COVID-19 patients and healthy volunteers demonstrate an increased CD45 expression on granulocytes (MFI and SEM; 36,252 ±1113 vs. 26,898 ± 2985; *p* = 0.0017) and on lymphocytes (165,919 ± 2715 vs. 119,202 ± 8970; *p* < 0.0001) from COVID-19 patients, but decreased expression on monocytes (134,756 ± 3536 vs. 209,266 ± 15,953; *p* < 0.0001). 

Incubation of whole blood samples from healthy volunteers with LPS revealed a marked 4.3-fold increase in CD45 expression on endotoxin-stimulated granulocytes from 26,898 ± 2985 to 114,144 ± 5293 (*p* < 0.0001), and a gradual, but highly significant, increase on lymphocytes from 119,202 ± 8970 to 158,107 ± 6564 (*p* = 0.0012) and monocytes from 209,266 ± 15,953 to 284,693 ± 11,880 (*p* = 0.0005). 

Incubation of whole blood samples from COVID-19 patients with LPS increased the CD45 expression on granulocytes from 36,252 ± 1113 to 93,807 ± 2012 (*p* < 0.0001) and on monocytes from 134,756 ± 3536 to 148,947 ± 3108 (*p* = 0.003), and reduced the expression on lymphocytes from 165,919 ± 2715 to 133,729 ± 2050 (*p* < 0.0001). 

LPS-induced CD45 expression was lower in COVID-19 patients than in healthy volunteers in all three investigated cell types, suggesting a downregulation of the Toll-like receptor-4-mediated signal transduction pathway in COVID-19, at least in respect to CD45.

**Figure 4:**
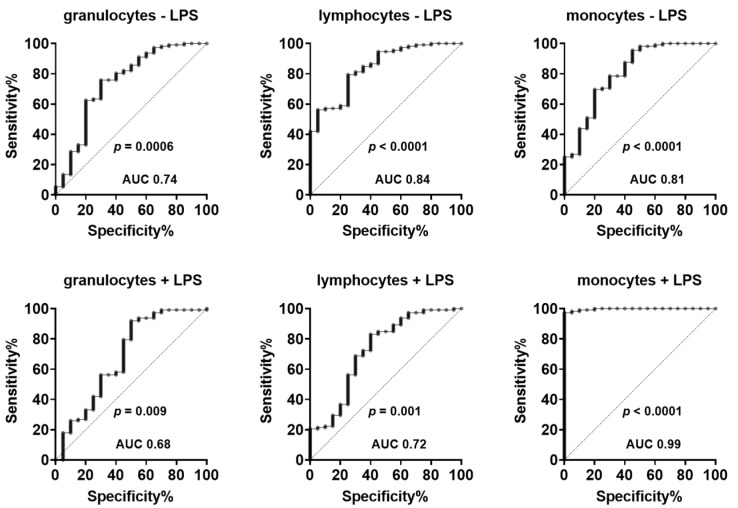
Receiver operating characteristic (ROC) curves describing the sensitivity and specificity of CD45 measurements in granulocytes, lymphocytes, and monocytes for the differentiation of healthy volunteers and COVID-19 patients. In the figures, the area under curve (AUC) and the significance levels are given.

To evaluate the eventual diagnostic value of CD45 to differentiate between healthy volunteers and patients with COVID-19, ROC curves were generated and AUC and asymptotic significance levels were determined. The results, shown in Figure 4, demonstrate that all CD45 measurements, both in absence and presence of LPS, allowed the differentiation of volunteers and COVID-19 patients with an AUC in the range from 0.68 to 0.99 (*p*: between 0.009 and 0.0001). The best differentiation between healthy volunteers and COVID-19 patients was achieved by measuring the surface expression of CD45 on monocytes after LPS stimulation with an AUC of 0.99 (*p* < 0.0001).

## 4. Discussion

The results of the present study demonstrate for the first time that the CD45 expression is altered in inflammation in a leucocyte subtype-specific manner, as evidenced in vitro in endotoxemia and in vivo in COVID-19 infection. The extent and direction of changes were dependent on the cell type investigated (granulocytes, lymphocytes, monocytes) and differed between short-term inflammatory stimulation with LPS and COVID-19 patients suggesting a complex regulation. CD45 can serve as a biomarker of inflammation, allowing the differentiation of healthy volunteers and COVID-19 patients. ROC ana-lysis demonstrated an excellent separation of groups with a maximum AUC of 0.99. The observed changes of CD45 in inflammation can be expected to be of physiological importance as the phosphatase is critically involved in the regulation of the immune system.

COVID-19 infection and LPS-exerted complex alterations of CD45 expression on leucocytes depend on both cell type and the inflammatory stimulus (lipopolysaccharide vs. COVID-19). CD45 expression in patients with COVID-19 was increased in comparison to healthy volunteers on granulocytes and lymphocytes, but was decreased on monocytes. The cellular response might be: (1) due to changes in signal transduction from binding to Toll-like receptor-4 and finally CD45 expression, (2) due to changes in leucocyte subtypes, or (3) due to maturation of cells. Moreover, LPS might cause the release of cytokines or other tissue hormones and indirect stimulation of other cell types. However, we cannot give any information on the exact mechanisms. Stimulation of whole blood samples from healthy volunteers with LPS markedly increased CD45 in granulocytes, lymphocytes, and monocytes. Stimulation of whole blood samples with LPS in patients with COVID-19 resulted in an increase in CD45 in granulocytes and monocytes, but not in lymphocytes. In vitro stimulation of leucocytes with LPS demonstrates that signal transduction events are involved in altered CD45 expression. However, no information on the mechanisms can be given.

The observed alterations in CD45 expression are an important finding, as the amount of the constitutively active protein tyrosine phosphatase is supposed to regulate the degree of dephosphorylation [10]. Moreover, pUL11, a viral protein and placental protein 14 (glycodelin), two ligands of CD45, have been demonstrated to be involved in the regulation of the phosphatase [10]. Increasing evidence indicates important physiological functions of CD45 in the regulation of both innate and adaptive immunity. Diseases spanning from severe combined immune defects, to susceptibility to viral infections, to autoimmune diseases are associated with CD45 [1,13,14]. Moreover, a downregulation of CD45 signaling in COVID-19 was described in peripheral blood mononuclear cells, which was reversed by the CD45-targeting peptide C24D [21]. However, no attempt was made to identify the exact cell type. In another study, CD45 activation with C24D reversed the tumor suppression of leucocyte function in breast cancer [18]. The results of the present study for the first time describe the modulation of CD45 surface expression upon inflammatory stimuli. This finding might be interpreted as an indicator for cellular reprogramming in inflammation. 

The present study has several limitations. We did not aim to determine changes in signal transduction, protein phosphorylation, or cellular function and further studies are needed to answer these questions. Moreover, we did not attempt to investigate the expression of CD45 subtypes, which might be worth investigating in further studies. A further technical note might derive from the fact that FACS analysis is rather difficult to standardize, as it is not easily possible to measure controls with standardized CD45 concentrations. In the present study, however, a standardized protocol with a single batch of antibodies, a new generation FACS analyzer, and daily quality checks were used. Moreover, changes in CD45 expression in absence and presence of LPS cannot be explained by such a “drift”. In addition, the finding that CD45 expression in COVID-19 patients increases in granulocytes and lymphocytes but decreases in monocytes cannot be explained by technical reasons, but reflects specific biological responses. 

## 5. Conclusions

The present study adds important knowledge to the physiology of CD45 in humans, demonstrating leucocyte subtype-specific alterations in the surface expression of the protein tyrosine phosphatase in COVID-19 and ex vivo endotoxemia. 

## Data Availability

Data can be provided on reasonable request.
